# Sodium–Glucose Transporter 2 Inhibitors in Heart Failure: An Overview of Systematic Reviews

**DOI:** 10.3390/jcdd11070198

**Published:** 2024-06-28

**Authors:** Yixuan Fang, Lihong Chen, Shiyi Sun, Xingwu Ran

**Affiliations:** 1 Department of Endocrinology & Metabolism, West China Hospital, Sichuan University, Chengdu 610041, China; f2216014698@163.com (Y.F.); chenlihong@scu.edu.cn (L.C.); sunshiyi2021@163.com (S.S.); 2 Innovation Center for Wound Repair, Diabetic Foot Care Center, West China Hospital, Sichuan University, Chengdu 610041, China

**Keywords:** Sodium–Glucose Transporter 2 inhibitors, heart failure, systematic review

## Abstract

**Background:** Several studies have shown that sodium-dependent glucose transporter 2 inhibitors can be used in the treatment of heart failure. This article summarized systematic reviews of sodium-dependent glucose transporter 2 inhibitors in the treatment of heart failure in order to evaluate efficacy and safety. **Methods:** We systematically searched eight electronic databases from inception to July 2023. We used Assessment of Multiple Systematic Reviews 2 to evaluate the methodological quality, the Preferred Reporting Items for Systematic Reviews and Meta-Analyses 2020 to assess report quality, Risk of Bias in Systematic Review to assess the risk of bias, and Grading of Recommendations Assessment, Development, and Evaluation to rate the quality of evidence. **Outcome:** A total of 36 systematic reviews were included. Our results were classified as clear evidence of benefit: hospitalization for heart failure; possible benefit: cardiovascular death (mortality) and renal outcome composite; clear evidence of no effect or equivalence: atrial arrhythmias, ventricular arrhythmia, atrial fibrillation, and hypotension; possible harm: genital infection; insufficient evidence to draw a conclusion: atrial flutter, major adverse cardiovascular events, urinary tract infection, acute kidney injury, hypoglycemia, and bone fracture. **Conclusions:** Sodium-dependent glucose transporter 2 inhibitors are beneficial for the treatment of heart failure, especially in terms of heart failure hospitalization.

## 1. Introduction

Heart failure (HF) is a clinical syndrome with dyspnea, fatigue, exercise intolerance, and fluid retention, which is a global health problem and affects at least 26 million people [1,2]. In the United States, the total number of people with HF increased by 46% from 2012 to 2023, more than 8 million people suffer from HF by 2023 and the prevalence of HF is predicted to increase by 23% [2,3]. In the meantime, medical costs are expected to grow from USD 30.7 billion to USD 69.8 billion and the quality of life of patients can only be improved by medical means such as drugs or implants [3,4].

As all we know, diabetes mellitus (DM) is an independent risk factor for HF, which was demonstrated in the Framingham study [5]. In the meantime, comparing with non-diabetics, people with diabetes have 2–4 times increased risk of cardiovascular disease [6]. Some studies recommend routinely checking blood urea nitrogen (BUN) and estimating glomerular filtration (eGFR) for all hospitalized patients with HF [7]. DM, HF, and diabetic nephropathy are a vicious circle, and any disease will lead to the other two [8].

Sodium-dependent glucose transporter 2 (SGLT-2) inhibitors, a new type of drug for the treatment of diabetes, are widely found in the kidneys and reabsorbs glucose through the glomeruli [9]. In many clinical trials, SGLT-2 inhibitors were beneficial for cardiovascular disease of diabetes and reduce the cardiovascular risk of diabetes [10,11]. Meanwhile, many studies suggested that SGLT-2 inhibitors can reduce the rate of cardiovascular hospitalization and mortality in HF patients [12,13,14]. As all we know, dapagliflozin and empagliflozin, as SGLT-2 inhibitors, serve as common drugs for treating DM, which can reduce glycated hemoglobin [15,16]. In 2021, European Society of Cardiology (ESC) guidelines recommended dapagliflozin and empagliflozin as Class I recommendations to reduce the risk of HF, hospitalization, and death in patients with HF with a preserved ejection fraction [17]. Although previous relevant articles have been published [18,19,20], this study aimed to update and refine previous findings. This study has different outcome indicators or included populations, and conducts a subgroup analysis on heart failure with a preserved ejection fraction or a reduced ejection fraction.

Overall, we focus on whether SGLT-2 inhibitors are effective and safe for patients with HF, in order to provide high-quality evidence-based medical evidence for clinicians, guideline development, and medication.

## 2. Methods

The study protocol was prospectively registered on the PROSPERO (CRD42023388117). This study refers to high-quality overviews of systematic reviews (SRs) [21,22].

### 2.1. Search Strategy

We systematically searched eight electronic databases from inception to July 2023, including Medline (PubMed), Cochrane Library, Embase, Scopus, Web of Science, the China Science and Technology Journal Database (CQVIP), Wanfang Data, and the Chinese National Knowledge Infrastructure (CNKI). The keywords, including “sodium–glucose cotransporter 2”, “dapagliflozin”, “canagliflozin”, “empagliflozin”, “ipragliflozin”, “sergliflozin”, “remokliflozin”, “totogliflozin”, “luseogliflozin”, “meta-analysis” and “systematic review” (SR), were used as the search keywords. To expand the search, we used intervention and study as search strategies. We had no language restrictions. To control for overlap in RCTs, we tried to select more studies from recent three years. We provided search queries for the English databases in Supplementary S1.

### 2.2. Eligibility Criteria

We presented the inclusion criteria through the PICOS principles. Additionally, there were no language restrictions, mainly English.

P (patient involvement): We included adults (>18) with a definite diagnosis of HF.

I (interventions): SGLT-2 Inhibitors were an intervention, independent of the type of drug.

C (control): The control group included a placebo, active controls, and standard treatment group.

O (outcomes): We used cardiovascular events and renal events as primary outcomes. Cardiovascular events were primarily defined as hospitalizations for heart failure (HHF), arrhythmia incidence, cardiovascular death (CVD), cardiovascular mortality, and major adverse cardiovascular events (MACE). We defined MACE as cardiovascular death, non-fatal myocardial infarction, and non-fatal stroke. Renal events included renal outcome composite. We defined renal outcome composite as estimated eGFR, end-stage renal disease, or renal death. In the meantime, adverse effects included urinary tract infections, hypoglycemia, hypotension, genital infections, bone fractures, and acute kidney injury (AKI).

S (study): We requested the inclusion of systematic reviews that included only randomized controlled trials (RCTs). In evidence-based medicine, randomized controlled trials were classified as the gold standard for the level of evidence [23]. We included only systematic reviews of RCTs because the evidence of RCTs is of higher quality and of greater clinical significance.

### 2.3. Study Selection

After importing the electronic database, duplicate documents would be deleted. Two independent reviewers (YXF, LHC) screened the titles and abstracts of the remaining literature. If there was a disagreement, it was resolved by the third reviewer (XWR).

### 2.4. Data Extraction

Two independent reviewers (YXF, SSY) extracted the data, and the third reviewer (XWR) resolved the disagreement. Six main types of data were extracted, including basic study characteristics (first author, year), outcomes (primary outcomes, number of included studies, number of patients), methodological characteristics (quality assessment tool), comparators (intervention, control measures), and patients baseline characteristics (heart failure status). At the same time, we recorded additional information on whether a meta-analysis was performed and whether the protocol was registered on the website.

### 2.5. Assessment of Methodological Quality

Two independent reviewers (YXF, LHC) assessed the methodological quality of the included systematic reviews using Assessment of Multiple Systematic Reviews 2 (AMSTAR 2). AMSTAR 2 is an updated version of AMSTAR that evaluates systematic reviews of randomized controlled trials [24]. Most current systematic reviews use AMSTAR 2 to assess methodological quality, which includes 16 questions. There are seven key projects, namely Q2, Q4, Q7, Q9, Q11, Q13, and Q15. The results of our assessment of methodological quality are based on these seven key questions [24]. We divide the results of each item into “Y”(each item met the criteria)”, PY (partially met the criteria), and “N”(not met the criteria at all).The final results are presented as high, moderate, low, and critically low. Disagreements would be resolved by the third reviewer (XWR).

### 2.6. Assessment of Reporting Quality

The reporting quality of systematic reviews were evaluated using the Preferred Reporting Items for Systematic Reviews and Meta-Analyses (PRISMA). PRISMA (2020) was from the PRISMA (2009) update. PRISMA (2020)consists of 27 entries, from title, abstract, method, and results to the comprehensive assessment of report quality [25]. The result of each item is evaluated as “Yes” (Y: fully reported),” Partial Yes” (PY: partially reported), and “No” (N: no reported). Two independent reviewers (YXF, LHC) assessed PRISMA (2020). Disagreements will be resolved by the third reviewer (XWR).

### 2.7. Assessment of Risk of Bias

Risk of Bias in Systematic Review (ROBIS) is a new tool [26]. The tool is completed in 3 stages to accurately assess the risk of bias. The three phases include assessing relevance, identifying concerns in the review process, and judging the risk of bias [26]. We independently assessed the risk of bias by two reviewers (YXF, LHC). Disagreements will be resolved by the third reviewer (XWR).

### 2.8. Assessment of Evidence Quality

Grading of Recommendations Assessment, Development, and Evaluation (GRADE) provides a transparent and structured process to evaluate the quality of evidence in SRs [27]. Two independent reviewers (YXF, LHC) assessed the evidence and a third reviewer (XWR) settled the disagreements. GRADE assesses the quality of evidence on five domains, including risk of bias, risk of inconsistency, risk of imprecision, indirect risk, and risk of publication bias. The results of the assessment were expressed as “high”, “moderate”, “low” and “critically low”.

### 2.9. Data Synthesis and Presentation

We mainly used a narrative description to analyze the included SRs. In the meantime, we did subgroup analyses. We analyzed each outcome by dividing it into two populations, including heart failure with a preserved ejection fraction (HFpEF) and heart failure with a reduced ejection fraction (HFrEF). In addition, we described and summarized the results. The data were mainly presented in the form of tables, including the basic characteristics of each systematic review, AMSTAR 2 results, ROBIS results, PRISMA results, GRADE results, and subgroup analyses.

We analyzed the outcomes and grouped them into the following categories. We referred to published articles for categorical definition [22,28]:

Clear evidence of benefit: the results of GRADE are moderate- or high-quality evidence and moderate- or high-quality evidence with confidence intervals (CIs) not crossing the line of no effect.

Clear evidence of harm: the results of GRADE are moderate- or high-quality evidence and moderate- or high-quality evidence with CIs not crossing the line of no effect.

Clear evidence of no effect or equivalence: moderate- or high-quality evidence with narrow CIs (approximately the range of risk ratio (RR) 0.75 to 1.25) crossing the line of no effect.

Possible benefit: low-quality evidence with a clear benefit, or moderate- or high-quality evidence with a wide CIs crossing the line no effect.

Possible harm: low-quality evidence with clear harm, or moderate or high-quality evidence with wide CIs crossing the line of no effect.

Insufficient evidence to draw a conclusion: low-quality evidence with wide CIs crossing the line of no effect or very low-quality evidence or evidence contradict each other.

## 3. Results

The basic characteristics of all systematic reviews were shown in Table S1 of Supplementary S3. All SRs were published between 2020 and 2023. The included populations and the types of research fully meet the criteria of the protocol. Among the 36 SRs [29,30,31,32,33,34,35,36,37,38,39,40,41,42,43,44,45,46,47,48,49,50,51,52,53,54,55,56,57,58,59,60,61,62,63,64], the largest number of included study was 17 RCT [62] and the lowest was 2 RCT [45]. Only five reviews did not evaluate the risk of bias using the Cochrane Collaboration risk of bias tool [33,44,45,47,62]. Only one systemic review provided a narrative description, while the rest provided statistical analyses. At the time of the PRISMA assessment, seven SRs were not analyzed for heterogeneity and nine SRs were not analyzed for sensitivity. The primary outcome of 31 systematic reviews were the cardiovascular outcomes. Only four SRs were missing.

### 3.1. Result of Literature Selection

**Figure 1:**
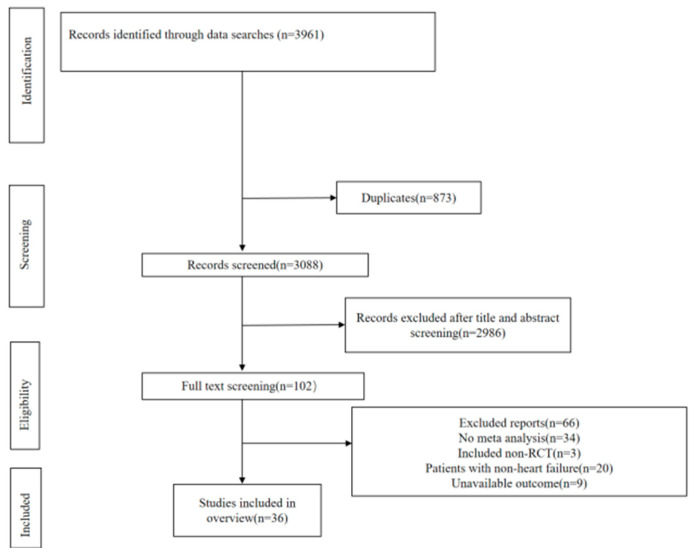
Study selection.

The process of screening studies is represented in Figure 1. There were 102 remaining articles exclude duplication and the title or abstract is not compliant. In the end, a total of 36 articles were selected after reading the full text. Excluded articles are in Supplementary S2.

### 3.2. Result of Methodological Quality (AMSTAR2)

We used AMSTAR2 to evaluate the methodological quality of included systematic reviews. One SR was rated as high quality,18 SRs as low quality, and 17 SRs as critically low quality. Seven key issues were as the probability of “Y”: Q2(33.3%), Q4(69.44%), Q7(5.56%), Q9(86.11%), Q11(94.45%), Q13(75.00%), and Q15(69.44%). Out of the 36 SRs, only two SRs were rated “Y” (Q7) in terms of providing a list of excluded studies and reasons for exclusions. This was the main reason for the low quality. Before starting to write the SR,15 SRs had registered the protocol on the website. In the 36 SRs, the type of selected studies were RCTs, but the reason for the inclusion of RCTs was not explained. The PICO principle was mentioned in 35 articles (Table S2 of Supplementary S4).

### 3.3. Result of Report Quality(PRISMA)

Overall, the quality of the report was good. There were four questions with 100% probability, including item 1, item 3, item 4, item 17. The advantages of the 36 SRs reports were as follows (probability of complete reports): (1) each review described the basic characteristics of the included studies (100%). (2) Effect measures (94.45%) and study selection (94.45%) were reported almost completely. The disadvantage of reduced reporting quality (probability of completely reporting): certainty assessment (11.11%) and certainty of evidence(13.89%) (Table S3 of Supplementary S5).

### 3.4. Result of Risk of Bias (ROBIS)

ROBIS tool is an assessment of the risk of bias in this review, and all results were expressed in Table S4 of Supplementary S6. In phase 1, there were 35 SRs which were rated as low risk. In phase 2, it is divided into three domains. Domain one, about the eligibility criteria for studies, had a low risk of 80.56%. Domain 2 had a minimum probability of low risk (27.8%), which was about the identification and selection of studies. Domain 3 and Domain 4 had similar low risk probabilities of 30.56% and 50%, respectively. In phase 3, 26 reviews were rated as low risk.

### 3.5. Result of Evidence Quality (GRADE)

We conducted GRADE on the outcomes which were consistent with all reviews, except for two articles (the one was not statistically analyzed [30] and the other was analyzed for different ejection fractions. We put it in a subgroup [63]). In addition, in the systematic evaluation by Zou et al., we only included adverse reactions and only included subgroup analysis for the main outcome indicators [37]. We classified all results according to GRADE grading. All results of the assessment were shown in Tables S5–S14 of Supplementary S7–S16, and all results were obtained in patients with heart failure who were given an SGLT-2 inhibitor versus a placebo (or standard treatment). At the same time, the results of the subgroup analysis were described (Tables S15 and S16 of Supplementary S17 and S18).

### 3.6. Cardiovascular Event

#### 3.6.1. HHF

Twenty-three SRs mentioned HHF. There were 14 SRs with high-quality evidence and 9 SRs with moderate-quality evidence. The odds ratio (OR)/risk ratio (RR)/hazard ratio (HR) ranged from 0.62 to 0.81. All confidence intervals did not cross the line of no effect. We consider the outcome as clear evidence of benefit. Overall, the reduced level of evidence was due to the risk of bias. We could assume that SGLT-2 inhibitors can reduce the risk of HHF in HF patients (Table S5 of Supplementary S7).

#### 3.6.2. Subgroup Analyses in HHF

There were six SRs mentioned HHF in HFrEF. All of the results suggested that SGLT-2 inhibitors can reduce the risk of hospitalization for heart failure in HFrEF patients.

Six SRs reported the HHF of HEpEF. One SR reported no difference in HHF results between SGLT-2 inhibitors and placebo (HR 0.726, 95%CI: 0.512, 1.028) [61]. The other five SRs suggested that SGLT-2 inhibitors are beneficial for HHF in HFpEF.

#### 3.6.3. Cardiovascular Death and Cardiovascular Mortality

Twenty SRs mentioned cardiovascular death. Seven SRs were rated high quality, and thirteen SRs were rated as moderate quality. The RR/HR/OR ranged from 0.72 to 0.96. The confidence intervals of six SRs crossed the line of no effect. Therefore, we classified the outcome as a possible benefit (Table S6 of Supplementary S8).

Cardiovascular mortality was mentioned in eight SRs. The RR/HR/OR ranged from 0.82 to 0.93. Only one SR crossed the line of no effect. We considered the outcome as a possible benefit (Table S6 of Supplementary S8).

In the meantime, there were two articles showing the results of all (first and recurrent) HHF or CVD. Additionally, two articles have shown that SGLT-2 inhibitors have a significant protective effect on all HHF or CVD [39,45].

Both cardiovascular death and cardiovascular mortality are the effects of SGLT-2 inhibitors on cardiovascular death events in patients with heart failure, and there is no essential difference.

#### 3.6.4. Subgroup Analyses in CVD

CVD of HFrEF was reported in eight SRs. Two SRs reported that SGLT-2 inhibitors were not associated with the occurrence of CVD (RR: 0.87, 95%CI: 0.74, 1.02; HR: 0.72, 95%CI: 0.42, 1.24) [37,48]. Six SRs showed that SGLT-2 inhibitors can reduce the risk of CVD in patients with HFrEF.

Ten SRs reported CVD in HEpEF. All of them showed that SGLT-2 inhibitors did not reduce the risk of CVD in HFpEF patients when comparing to placebo.

#### 3.6.5. Arrhythmia

There were two SRs which mentioned atrial fibrillation. The range of RR was from 0.62 to 0.92 [36,64]. One SR was rated high quality, and the other SR was rated moderate quality with narrow CIs which crossed the line of no effect. We considered the outcome as clear evidence of no effect or equivalence.

There was one SR who mentioned ventricular arrhythmia and atrial arrhythmias [44]. Additionally, ventricular arrhythmia and atrial arrhythmias was evaluated as moderate/high quality with a narrow CIs crossing the line with no effect. We considered that ventricular arrhythmia and atrial arrhythmias could be rated as clear evidence of no effect or equivalence.

However, the result of the evaluation of atrial flutter was low-quality evidence with wide confidence intervals in HFrEF [64] We thought the outcome was insufficient evidence to draw a conclusion (Table S7 of Supplementary S9).

#### 3.6.6. Subgroup Analyses in Arrhythmia

Two SRs reported atrial fibrillation in HFrEF [36,64]. (RR 0.8, 95%CI: 0.63, 1.02; RR0.62, 95%CI: 0.44, 0.86) Only one SR reported atrial fibrillation in HFpEF [36]. (RR 0.99, 95%CI: 0.83, 1.17) We could not obtain definite conclusions about the effect of the ejection fraction on arrhythmia.

#### 3.6.7. MACE

Only two SRs reviewed MACE. We only selected the MACE outcome indicators which were consistent with the definition in this article.HR are 0.81, 0.95, respectively [33,39]. However, the confidence interval included 1 and all GRADE results were of low quality. We considered the outcome was insufficient evidence to draw a conclusion (Table S8 of Supplementary S10).

### 3.7. Renal Event

Only two SRs assessed renal outcome composite, one with high-quality evidence (HR 0.60, 95%CI: 0.48, 0.75) [52] and the other with low-quality evidence (HR 0.62, 95%CI: 0.43, 0.90) [45]. We thought the outcome could be possibly beneficial (Table S9 of Supplementary S11).

As for subgroups, three SRs reported composite renal outcomes in patients with HFrEF. Some studies suggested that SGLT-2 inhibitors were beneficial for composite kidney outcomes (HR 0.62, 95%CI: 0.43, 0.9; HR 0.63, 95%CI: 0.43, 0.91) [45,52]. However, Zou et al. thought that there was no difference between SGLT-2 inhibitors and placebo in terms of composite kidney outcome (RR 0.6, 95%CI: 0.08, 4.68) [37].

Only one SR reported the renal outcome composite for HEpEF. Zou et al. reported that in patients with HEpEF patients, there was no difference in renal outcome composite between SGLT-2 inhibitors and placebo (RR 0.96, 95%CI: 0.74, 1.25) [37].

### 3.8. Adverse Effects

#### 3.8.1. Urinary Tract Infections

Four SRs analyzed data on urinary tract infections. Two of them were of low quality and two were rated as moderate quality. The RR ranged from 0.82 to 1.131. Cao et al. found that SGLT-2 inhibitors increased the risk of urinary tract infections when comparing with placebo [39]. However, the other SRs found no effect. We considered this result was insufficient evidence to draw a conclusion (Table S10 of Supplementary S12).

#### 3.8.2. Genital Infections

A total of six SRs assessed the quality of evidence for genital infections. There were three high-quality, two moderate- and one low-quality. RR/OR was greater than 1. The main reason of reducing the quality of evidence was the risk of bias. We thought the results might be rated as possible harm (Table S11 of Supplementary S13).

#### 3.8.3. Hypoglycemia and Hypotension

Nine SRs evaluated the quality of evidence for hypoglycemia. There were five low-quality evidence SRs, and four moderate-quality evidence SRs. All RR/OR ranged from 0.69 to 1.14. We considered the result was insufficient to draw conclusions (Table S12 of Supplementary S14).

Four SRs mentioned hypotension which included three moderate-quality evidence SRs and one high-quality evidence SR. The range of RR was from 1.09 to 1.17. We considered the hypotension as clear evidence of no effect or equivalence (Table S12 of Supplementary S14).

#### 3.8.4. Bone Fracture

Here, were seven SRs with meta-analysis for bone fracture. There were four of moderate quality, two low quality, and one of very low quality. The range of RR/OR was more than 1. However, all of the confidence intervals cross the line of no effect. We considered the outcome could be insufficient evidence to draw a conclusion (Table S13 of Supplementary S15).

#### 3.8.5. Acute Kidney Injury

There were eight SRs, including two with high-quality evidence, one moderate-quality evidence, and five low-quality evidence. All HR/RR/OR were less than 1. At the same time, two SRs suggested that SGLT-2 inhibitors could reduce the occurrence of AKI [41,50]. However, the others found that SGLT-2 inhibitors did not aggravate the occurrence of AKI. Therefore, we considered the results insufficient evidence to draw a conclusion (Table S14 of Supplementary S16).

#### 3.8.6. Subgroup Analyses in Adverse Effects

Two SRs reported the data about adverse effects of HFrEF. SGLT-2 inhibitors might increase the risk of genital infection (RR: 2.09, 95%CI: 1.26, 3.47; RR: 2.32, 95%CI: 1.26, 4.27) [37,43]. There was no difference in other adverse effects between SGLT-2 inhibitors and placebo.

Only one SRs reported about adverse events in HFpEF. They found that SGLT-2 inhibitors were more likely to cause genital infections than placebo (RR: 3.04, 95%CI: 1.88, 4.91) [37].

## 4. Discussion

Compared to the previous review [18], we included more studies, more outcome indicators, and a more comprehensive analysis of the results. Compared with the other two articles, due to the differences in the included population and analysis methods, the connection between SGLT-2 inhibitors and heart failure patients is elaborated from different perspectives [19,20]. This study found, through a comprehensive evaluation of the systematic reviews of SGLT-2 inhibitors in patients with heart failure, benefits in cardiovascular disease outcomes, possibly related to the following mechanisms. SGLT-2 inhibitor can reduce the symptoms of congestion in heart failure. By reducing sodium and glucose reabsorption, the absorption of water decreases, indirectly leading to reduced sodium reabsorption and increased electrolyte-free water excretion [65]. Empagliflozin can improve myocardial function, increase left ventricular systolic function, and improve adverse left ventricular remodeling [66]. In the meantime, empagliflozin can improve hemodynamics and myocardial fibrosis [67]. SGLT-2 inhibitors can reduce glomerular hyperfiltration caused by hyperglycemia and alleviate kidney damage [68]. SGLT-2 inhibitors may inhibit sodium and potassium pumps, reduce ATP consumption and environmental hypoxia, improve hematopoietic and hematocrit, and alleviate chronic kidney damage [69].

According to the European Society of Cardiology (ESC) guidelines for angiotensin-converting enzyme (ACEI), mineralocorticoid receptor antagonist (MRA), and β receptor blocker, drugs are recommended for the treatment of heart failure with a reduced ejection fraction [70]. The advantage of SGLT-2 inhibitors over previous drug therapies is that they are used as a treatment for diabetes and have benefits for patients with chronic kidney disease [71]. Patients with heart failure often coexist with diabetes and chronic kidney disease and can form a vicious cycle [8]. The use of SGLT-2 inhibitors can maximize the drug efficacy and can be used as a multidisciplinary drug regimen for patients with heart failure comorbidities.

## 5. Conclusions

First of all, we think that SGLT-2 inhibitors are beneficial for HHF. CVD and cardiovascular mortality are considered as evidence of a possible benefit. However, in subgroup analysis, SGLT-2 inhibitors do not differ in terms of CVD or HFpEF when comparing with placebo. Regarding atrial arrhythmias and atrial fibrillation, there was no clear evidence of effect or equivalence. In the meantime, atrial flutter indicated insufficient evidence to draw a conclusion. However, ventricular arrhythmia was rated as a possible benefit. As for renal outcomes, we classify renal outcome composite as a possible benefit in HF. However, we found no difference in renal outcome composite in HFpEF patients with SGLT-2 inhibitors compared to placebo. Therefore, more clinical trials are needed. Regarding adverse reactions, we found that SGLT-2 inhibitors may increase the incidence of genital infection in HF patients, and the results were consistent in subgroup analyses.

Secondly, methodological quality was low when assessed using the AMSTAR-2 tool. This is mainly because there was no reason to provide a list of excluded studies and justify exclusions (Q7). The GRADE tool was used to grade the quality of the outcome evidence. In Risk of Bias in Systematic Review (ROBIS), the lowest risk of bias appeared during the process of searching and screening. In the GRADE tool, the main reason for the reduction in outcome indicators is the risk of bias in the included RCTs.

Lastly, according to the evaluation of SR in this paper, we summarized a few points that require attention when writing SRs. Primarily, SRs should be written strictly according to PRISMA 2020, register the systematic review protocol in advance, make appropriate use of existing tools to evaluate the included studies, and transparency is needed in the screening process.

## 6. Limitations

The limitations of this study are mainly shown in the following aspects. Firstly, there was an overlap in the RCTs included in the reviews. However, we tried to select more recent literature to avoid overlap. Secondly, in this study, the drugs included in SGLT-2 inhibitors were not carefully distinguished, so we referred to them collectively as SGLT-2 inhibitors. This may make clinical medication guidance difficult. Thirdly, we did not search for grey literature, which may lead to bias in the search process.

## Data Availability

Data are contained within this article or Supplementary Material.

## Supplementary Materials

The following supporting information can be downloaded at: https://www.mdpi.com/article/10.3390/jcdd11070198/s1, S1—Search strategy. S2—List of excluded documents. S3—Table S1: The basic characteristics of all systematic reviews. S4—Table S2: The results of AMSTAR2. S5—Table S3: The result of PRISMA. S6—Table S4: The result of ROBIS. S7—Table S5: HHF result in GRADE. S8—Table S6: CVD and Cardiovascular Mortality result in GRADE. S9—Table S7: Result of Evidence Quality (GRADE). S10—Table S8: Result of Evidence Quality (GRADE). S11—Table S9: Result of Evidence Quality (GRADE). S12—Table S10: Result of Evidence Quality (GRADE). S13—Table S11: Result of Evidence Quality (GRADE). S14—Table S12: Result of Evidence Quality (GRADE). S15—Table S13: Result of Evidence Quality (GRADE). S16—Table S14: Result of Evidence Quality (GRADE). S27—Table S15: Subgroup analyses in HFrEF. S18—Table S16: Subgroup analyses in HFpEF.
