# Supplementary material for: Sodium–Glucose Transporter 2 Inhibitors in Heart Failure: An Overview of Systematic Reviews
Source: J Cardiovasc Dev Dis. 2024 Jun 28;11(7):198. doi: 10.3390/jcdd11070198 (PMC11276734; doi:10.3390/jcdd11070198)
Supplement: Supplementary file 1 [file jcdd-11-00198-s001.zip › S1.pdf]

Search strategy

PUBMED:

#1 "Sodium-Glucose Transporter 2 Inhibitors"[MeSH Terms]
#2 "dapagliflozin"[Supplementary Concept]
#3"Canagliflozin"[Title/Abstract]
#4 "empagliflozin"[Title/Abstract]
#5 "ertugliflozin" [Title/Abstract]
#6 "ipragliflozin"[Title/Abstract]
#7 "sergliflozin"[Title/Abstract]
#8 "remogliflozin"[Title/Abstract]
#9 "tofogliflozin"[Title/Abstract]
#10 "luseogliflozin"[Title/Abstract]
#11 #1 OR #2 OR #3 OR #4 OR #5 OR #6 OR #7 OR#8 OR #9 OR #10
#12 "meta analysis"[Title/Abstract]
#13 "systematic review"[Title/Abstract]
#14 “systematic”
#15 #12 OR #13 OR #14
#16 #11 AND #15

Ovid MEDLINE:

1 Sodium-Glucose Transporter 2 Inhibitors
2 dapagliflozin.mp.
3 Canagliflozin/
4 empagliflozin.mp.
5 ertugliflozin.mp.
6 ipragliflozin.mp.
7 sergliflozin.mp.
8 remogliflozin.mp.
9 tofogliflozin.mp.
10 luseogliflozin.mp.
11 Meta-Analysis/
12 "Systematic Review"/
13 1 or 2 or 3 or 4 or 5 or 6 or 7 or 8 or 9 or 10
14 11 or 12
15 13 and 14

Web Of Science: (TS=(Sodium-Glucose Transporter 2 Inhibitors or dapagliflozin or Canagliflozin or empagliflozin or ertugliflozin or ipragliflozin or sergliflozin or remogliflozin or tofogliflozin or luseogliflozin)) AND TS=(meta analysis or systematic review)

Cochrane Library

#1(Sodum Glucose Transporter 2 nhibtors or SCLT-2 inhibitors or SGLT 2 nhibitors or SCLT2 nhibitors or Sodum-Glucose Transporter 2 inhibitor or SodiumGlucose Transporter 2 lnhibitor or SGLT2 lnhibitor or lnhibitor, SGLT2 or Glozins or Glfozin or SGLT-2 nhibitor or nhibior, SGLT-2 0r SGLT 2 lnhibitor)xt,ab,kw
#2(dapagliflozin):ti,ab,kw

#3(Canagliozin or empagliflozin or ertugliflozin or ipragliflozin or serglifozin or remoglifozin or tofogliflozin or luseogliflozin):ti,abkw
#4(systematic review):ti,ab,kw
#5 #1 or #2 or #3
#6(meta-analysis"):ti,ab.kw
#7 #4 or #6
#8 #5 AND #7
Embase
1 sodium glucose cotransporter 2 inhibitor/
2 dapagliflozin/
3 canagliflozin/
4 empagliflozin/
5 ertugliflozin/
6 ipragliflozin/
7 remogliflozin.mp.
8 tofogliflozin/
9 luseogliflozin/
10 meta analysis/
11 "systematic review"/
12 sergliflozin.mp.
13 heart failure/
14 1 or 2 or 3 or 4 or 5 or 6 or 7 or 8 or 9 or 12
15 10 or 11
16 13 and 14 and 15
Scopus
(TITLE-ABS-KEY ("sodium-glucose transporter 2 inhibitors" OR dapagliflozin OR canagliflozin OR empagliflozin OR ertugliflozin OR ipragliflozin OR sergliflozin OR remogliflozin OR tofogliflozin OR luseogliflozin) AND TITLE-ABS-KEY ("meta analysis" OR "systematic review")) AND (LIMIT-TO (DOCTYPE , "re") OR LIMIT-TO (DOCTYPE , "ar"))
