# Supplementary material for: Sodium–Glucose Transporter 2 Inhibitors in Heart Failure: An Overview of Systematic Reviews
Source: J Cardiovasc Dev Dis. 2024 Jun 28;11(7):198. doi: 10.3390/jcdd11070198 (PMC11276734; doi:10.3390/jcdd11070198)
Supplement: Supplementary file 1 [file jcdd-11-00198-s001.zip › S10.pdf]

Table S8 Result Of Evidence Quality （GRADE）

Outcome	Review	Certainty assessment							Certainty
		Relative effect（95%CI）	studies（sample size）	Risk of bias	Inconsistency	Indirectness	Imprecision	Other considerations	
MACE	Kumar et al,2020[33]	HR 0.81（0.59,1.10）	3	not serious	serious(f)	not serious	serious(g)	not serious	low
MACE	Cao et al,2022[39]	HR 0.95（0.83,1.09）	4	serious(c)	not serious	not serious	serious(g)	not serious	low

MACE: major adverse cardiovascular events; c: selective reporting bias; f: I^2>50%; g: confidence interval contains Invalid line; HR: hazard rate
