# Supplementary material for: Sodium–Glucose Transporter 2 Inhibitors in Heart Failure: An Overview of Systematic Reviews
Source: J Cardiovasc Dev Dis. 2024 Jun 28;11(7):198. doi: 10.3390/jcdd11070198 (PMC11276734; doi:10.3390/jcdd11070198)
Supplement: Supplementary file 1 [file jcdd-11-00198-s001.zip › S11.pdf]

Table S9 Result Of Evidence Quality （GRADE）

Outcome	Review	Certainty assessment							Certainty
		Relative effect（95%CI）	Studies（sample size）	Risk of bias	Inconsistency	Indirectness	Imprecision	Other considerations	
Renal outcome composite	Zannad et al,2020[45]	HR 0.62（0.43,0.90）	2（118）	not serious	serious(e)	not serious	serious(h)	not serious	low
Renal outcome composite	Butler et al,2020[52]	HR 0.60（0.48,0.75）	4	not serious	not serious	not serious	not serious	not serious	High

e: poor overlap of confidence interval; h: sample size is too small; HR: hazard rate
