# Supplementary material for: Sodium–Glucose Transporter 2 Inhibitors in Heart Failure: An Overview of Systematic Reviews
Source: J Cardiovasc Dev Dis. 2024 Jun 28;11(7):198. doi: 10.3390/jcdd11070198 (PMC11276734; doi:10.3390/jcdd11070198)
Supplement: Supplementary file 1 [file jcdd-11-00198-s001.zip › S12.pdf]

Table S10 Result Of Evidence Quality （GRADE）

Outcome	Systematic review	Certainty assessment							Certainty
		Relative effect （95%CI）	studies（patiens）	Risk of bias	Inconsistency	Indirectness	Imprecision	Other considerations	
urinary tract infection	Cao et al,2022[39]	RR 1.17(1.03,1.33)	4	serious(c)	not serious	not serious	not serious	not serious	Moderate
urinary tract infection	Wu et al,2022[43]	RR 1.06 （0.84,1.37）	5（226）	serious(bd)	not serious	not serious	serious(g)	not serious	Low
urinary tract infection	Li et al,2021[53]	RR1.131(0.897,1.426)	2	not serious	not serious	not serious	serious(g)	not serious	Moderate
urinary tract infection	Cai et al,2021[59]	RR 0.82 （0.43,1.57）	3（38）	serious(c,d)	not serious	not serious	serious(g)	not serious	Low

b: bias of blind method; c: Selective reporting bias; d: incomplete data results; g: confidence interval contains invalid line; RR: relative risk; HR: hazard rate
