# Supplementary material for: Sodium–Glucose Transporter 2 Inhibitors in Heart Failure: An Overview of Systematic Reviews
Source: J Cardiovasc Dev Dis. 2024 Jun 28;11(7):198. doi: 10.3390/jcdd11070198 (PMC11276734; doi:10.3390/jcdd11070198)
Supplement: Supplementary file 1 [file jcdd-11-00198-s001.zip › S13.pdf]

TableS11 Result Of Evidence Quality （GRADE）

Outcome	Systematic review	Certainty assessment							Certainty
		Relative effect （95%CI）	studies（patiens）	Risk of bias	Inconsistency	Indirectness	Imprecision	Other considerations	
Genital infection	Zou et al,2022[37]	RR 2.69 (1.61,4.52)	5(143）	not serious	not serious	not serious	not serious	not serious	High
Genital infection	Li et al,2021[53]	RR 2.772 (1.474,5.212)	2	not serious	not serious	not serious	not serious	not serious	High
Genital infection	Shrestha et al,2021[38]	OR 2.29 （1.33,3.92）	5 （62）	serious(a)	not serious	not serious	not serious	not serious	Moderate
Genital infection	Wu et al,2022[43]	RR 2.09 （1.26,3.47）	4（65）	serious(bd)	serious(e)	not serious	not serious	not serious	low
Genitalia infection	Bazoukis et al,2021[50]	RR 1.9 （0.34,10.45）	4	not serious	not serious	not serious	serious(j)	not serious	Moderate
Genital infection	Younes et al,2022[47]	OR 2.97 （2.02,4.36）	3（138）	not serious	not serious	not serious	not serious	not serious	High

a: included RCT has risk of bias; b: bias of blind method; b: bias of blind method; d :incomplete data results ;e: poor overlap of confidence interval ;j: the confidence interval is too wide; OR: odds ratio; RR: relative risk
