# Supplementary material for: Sodium–Glucose Transporter 2 Inhibitors in Heart Failure: An Overview of Systematic Reviews
Source: J Cardiovasc Dev Dis. 2024 Jun 28;11(7):198. doi: 10.3390/jcdd11070198 (PMC11276734; doi:10.3390/jcdd11070198)
Supplement: Supplementary file 1 [file jcdd-11-00198-s001.zip › S14.pdf]

Table S12 Result Of Evidence Quality （GRADE）

Outcome	Systematic review	Certainty assessment							Certainty
		Relative effect （95%CI）	studies（patiens）	Risk of bias	Inconsistency	Indirectness	Imprecision	Other considerations	
Hypoglycemia	Kumar et al,2020[33]	HR 1（0.29,3.45）	2	not serious	not serious	not serious	serious(j)	not serious	Moderate
Hypoglycemia	Shrestha et al,2021[38]	OR 1.14 （0.81,1.61）	8(133)	serious(a)	not serious	not serious	serious(g)	not serious	low
Hypoglycemia	Cao et al,2022[39]	RR 0.95(0.8,1.14)	5	serious(c)	not serious	not serious	serious(g)	not serious	low
Hypoglycemia	Wu et al,2022[43]	RR 0.93(0.61,1.42)	5(84)	serious(bd)	not serious	not serious	serious(g)	not serious	low
Hypoglycemia	Younes et al,2022[47]	OR 1.02（0.8,1.31）	4（257）	not serious	not serious	not serious	serious(g)	not serious	Moderate
Hypoglycemia	Zheng et al,2020[54]	RR 1.09(0.78,1.53)	3(103)	serious(d)	not serious	not serious	serious(g)	not serious	low
Hypoglycemia	Bazoukis et al, 2021[50]	RR 0.93 （0.75,1.16）	6	not serious	not serious	not serious	serious(g)	not serious	Moderate
Hypoglycemia	Cai et al,2021[59]	RR 0.69（0.34,1.4）	4（35）	serious(c,d)	not serious	not serious	serious(g)	not serious	low
Hypoglycemia	Li et al,2021[53]	RR1.136(0.875,1.391)	4	not serious	not serious	not serious	serious(g)	not serious	Moderate
Hypotension	Vukadinovi´c et al,2020[41]	RR 1.09 （0.91,1.31）	3(421)	not serious	not serious	not serious	serious(g)	not serious	Moderate
Hypotension	Younes et al,2022[47]	RR 1.17 (1.03,1.33)	4	not serious	not serious	not serious	not serious	not serious	High
Hypotension	Bazoukis et al,2021[50]	RR 1.09（0.9,1.31）	4(989)	not serious	not serious	not serious	serious(g)	not serious	Moderate
Hypotension	Li et al,2021[53]	RR 1.112 (0.922,1.340)	4	not serious	not serious	not serious	serious(g)	not serious	Moderate

a: included RCT has risk of bias; c: selective reporting bias; b: bias of blind method; d: incomplete data results; e: poor overlap of confidence interval ; g: confidence interval contains Invalid line; j: the confidence interval

is too wide; OR: odds ratio; RR: relative risk; HR: hazard rate
