# Supplementary material for: Sodium–Glucose Transporter 2 Inhibitors in Heart Failure: An Overview of Systematic Reviews
Source: J Cardiovasc Dev Dis. 2024 Jun 28;11(7):198. doi: 10.3390/jcdd11070198 (PMC11276734; doi:10.3390/jcdd11070198)
Supplement: Supplementary file 1 [file jcdd-11-00198-s001.zip › S15.pdf]

Table S13 Result Of Evidence Quality （GRADE）

Outcome	Systematic review	Certainty assessment							Certainty
		Relative effect （95%CI）	studies（patiens）	Risk of bias	Inconsistency	Indirectness	Imprecision	Other considerations	
Bone fracture	Shrestha et al,2021[38]	OR 1.15 （0.94,1.41）	6（384）	serious(a)	not serious	not serious	serious(g)	not serious	Low
Bone fracture	Zou et al,2022[37]	RR 1.05(0.81,1.36)	5（450）	not serious	not serious	not serious	serious(g)	not serious	Moderate
Bone fracture	Cao et al,2022[39]	RR 1.04 （0.88,1.23）	6	serious(c)	serious(e)	not serious	serious(g)	not serious	Very low
Bone fracture	Wu et al,2022[43]	RR 1.06 （0.82,1.36）	4（230）	serious(bd)	not serious	not serious	serious(g)	not serious	Low
Bone fracture	Cardoso et al,2021[46]	OR 1.09(0.85,1.4)	6 (252)	not serious	not serious	not serious	serious(g)	not serious	Moderate
Bone fracture	Li et al,2021[53]	RR 1.104(0.875,1.391)	4	not serious	not serious	not serious	serious(g)	not serious	Moderate
Bone fracture	Younes et al,2022[47]	OR 1.06(0.88,1.27)	4(467)	not serious	not serious	not serious	serious(g)	not serious	Moderate

a: included RCT has risk of bias; b: bias of blind method; c: selective reporting bias; d: incomplete data results; g: confidence interval contains invalid line; OR: odds ratio; RR: relative risk
