# Supplementary material for: Sodium–Glucose Transporter 2 Inhibitors in Heart Failure: An Overview of Systematic Reviews
Source: J Cardiovasc Dev Dis. 2024 Jun 28;11(7):198. doi: 10.3390/jcdd11070198 (PMC11276734; doi:10.3390/jcdd11070198)
Supplement: Supplementary file 1 [file jcdd-11-00198-s001.zip › S16.pdf]

Table S14 Result Of Evidence Quality （GRADE）

Outcome	Review	Certainty assessment							Certainty
		Relative effect （95%CI）	Studies（sample size）	Risk of bias	Inconsistency	Indirectness	Imprecision	Other considerations	
AKI	Shrestha et al,2021[38]	OR 0.73（0.47, 1.14）	5（131）	serious(a)	not serious	not serious	serious(g)	not serious	low
AKI	Cao et al,2022[39]	RR 0.89(0.78,1.01)	5	serious(c)	not serious	not serious	serious(g)	not serious	low
AKI	Kumar et al,2020[33]	HR 0.89(0.72,1.11)	3	not serious	not serious	not serious	serious(g)	not serious	Moderate
AKI	Vukadinovi´c et al, 2020[41]	RR 0.69 （0.51,0.93）	4（235）	not serious	not serious	not serious	not serious	not serious	High
AKI	Wu et al ,2022[43]	RR 0.87 （0.73,1.03）	4（498）	serious(bd)	not serious	not serious	serious(g)	not serious	low
AKI	Younes et al, 2022[47]	OR 0.81 （0.58,1.12）	4 （878）	not serious	serious(f)	not serious	serious(g)	not serious	low
AKI	Bazoukis et al, 2021[50]	RR 0.67 （0.52,0.87）	6	not serious	not serious	not serious	not serious	not serious	High
AKI	Li et al,2021[53]	RR 0.74 （0.45,1.19）	3	not serious	serious(e)	not serious	serious(g)	not serious	low

AKI: Acute kidney injury; a: included RCT has risk of bias; b:bias of blind method ;c: selective reporting bias ;d: incomplete data results ;e: poor overlap of confidence interval ;f: I^2>50% or the heterogeneity test P was very small ;g: Confidence interval contains Invalid line; OR: odds ratio; RR: relative risk; HR: hazard rate
