# Supplementary material for: Sodium–Glucose Transporter 2 Inhibitors in Heart Failure: An Overview of Systematic Reviews
Source: J Cardiovasc Dev Dis. 2024 Jun 28;11(7):198. doi: 10.3390/jcdd11070198 (PMC11276734; doi:10.3390/jcdd11070198)
Supplement: Supplementary file 1 [file jcdd-11-00198-s001.zip › S17.pdf]

Table S15 Subgroud analyses in HFrEF

patient	systematic review	Patient number in intervention(n1)/control(n2), included studies(n) and Statistics of outcome
HFrEF	Zou et al,2020[37]	HHF RR 0.72,95%CI(0.6,0.86),(n1=478,n2=663);CVD RR 0.87,（0.74,1.02）,n1=415,n2=479;renal outcome composite RR 0.6,CI(0.08,4.68),n1=58,n2=97; bone fracture RR 1.03, 95%CI(0.74,1.43) ,n1=95,n2=92; genital infection RR 2.32, 95%CI(1.26,4.27),n1=38,n2=16
	Vaduganathan et al,2020[42]	CVD HR 0.86,95%CI(0.76,0.98),n1=414,n2=475; HHF HR 0.69,95%CI(0.62,0.78),n1=477,n2=660;
	Wu et al,2020[43]	HHF HR0.7,95%CI（0.62,0.78),n=4;CVD HR 0.84,95%CI (0.74,0.95),p=0.006,n=4; genital infection RR 2.09,95%CI（1.26,3.47）n1=44,n2=21,n=4; urinary tract infection RR 1.06,95%CI（0.98,1.37）, n1=116,n2=110,n=5; hypoglycemia RR 0.93,95%CI（0.61,1.42）, n1=40,n2=44,n=5; bone fracture RR 1.06,95%CI（0.82,1.36）, n1=117,n2=113;AKI RR0.87,95%CI(0.73,1.03),n1=231,n2=267,n=4
	Zannad et al,2020[45]	CVD HR0.86,95%CI(0.76,0.98),n1=414,n2=475,n=2;renal outcome composite HR 0.62,95%CI(0.43,0.9),n1=46 n2=72, n=2;
	Tsampasian et al,2021[48]	CVD HR0.72,95%CI (0.42,1.24),n1=67,n2=68,n=2;
	Butler et al,2020[52]	CVD HR O.83,95%CI(0.71,0.98),n=4; renal outcome composite HR 0.63,95%CI(0.43,0.91),n=2;
	Zhai et al,2021[55]	HHF OR 0.74,95%CI(0.6,0.91),n1=186,n2=244,n=4,CVD OR 0.72,95%CI（0.58,0.91）,n1=149,n2=202,n=4
	Ouyang etal,2023[36]	atrial fibrillation RR0.8（0.63,1.02),n=112,n2=140
	Singh et al,2021[61]	HHF HR 0.686,95%CI(0.615,0.785);CVD HR 0.842,95%CI（0.744,0.953)
	Chen et al,2022[60]	HHF HR 0.69,95% CI (0.62,0.76)
	Sfairopoulos et al,2022[64]	atrial fibrillation RR0.62,95%CI(0.44,0.86),n1=54,n2=88,n=6;atrial flutter ,RR 0.85,95%CI（0.09,7.92),n1=11,n2=14,n=2;

HFrEF: heart failure with reduced ejection fraction; HHF: Hospitalization for heart failure; CVD: cardiovascular death；RR: relative risk; HR: hazard rate
