# Supplementary material for: Sodium–Glucose Transporter 2 Inhibitors in Heart Failure: An Overview of Systematic Reviews
Source: J Cardiovasc Dev Dis. 2024 Jun 28;11(7):198. doi: 10.3390/jcdd11070198 (PMC11276734; doi:10.3390/jcdd11070198)
Supplement: Supplementary file 1 [file jcdd-11-00198-s001.zip › S18.pdf]

Table S16 Subgroud analyses in HFpEF

Patient	Review	Patient number in intervention(n1)/control(n2), included studies(n) and Statistics of outcome
HFpEF	Fukuta,et al,2022[35]	HHF OR 0.714 95%CI(0.612,0.831),n=4;CVD OR 0.949 95%CI(0.8,1.126),n=3
	Zou et al,2022[37]	HHF RR0.73,95%CI(0.61,0.88),n1=260,n2=353;CVD RR0.9, 95%CI(0.29,2.79), n1=219,n2=244;composite kidney outcome RR0.96, 95%CI(0.74,1.25),bone fracture RR 1.07, 95%CI(0.51,2.24), n1=136, n2=127;genital infection RR 3.04, 95%CI(1.88,4.91),n1=67,n2=22
	Cao et al,2022[39]	CVD HR 0.99,95%CI (0.84,1.15),n=3
	Vaduganathan et al,2022[42]	CVD HR 0.88,95%CI(0.77,1),n1=417,n2=474, HHF HR 0.74 ,95%CI (0.67,0.83),n1=588,n2=770;
	Tsampasian et al,2021[48]	CVD HR 1.27,95%CI(0.92,1.76),N1=101,N2=59,N=2;
	Butler et al,2020[52]	CVD HR 1.27,95%CI(0.92,1.76),N=2;
	Zhai et al,2021[55]	CVD OR 1.45 95%CI（0.95,2.22）, n1=56,n2=39,n=2
	Zhou et al,2022[57]	CVD HR 0.96 ,95%CI(0.82,1.13),n=3
	Ouyang et al, 2023[36]	atrial fbrillation RR 0.99(0.83,1.17), n1=236,n2=239,n=4
	Chen et al, 2022[60]	HFH HR 0.74(0.67,0.82)
	De Marzo et al,2023[51]	CVD=RR 0.94(0.85,1.04),N=5;HFH RR 0.81(0.73,0.9),N=5
	Singh et al,2021[61]	HHF HR 0.726,95%CI(0.512,1.028),N=2,CVD HR 1.236, 95%CI(0.848,1.801), N=2

HFpEF: heart failure with preserved ejection fraction; CVD: cardiovascular death; HHF: Hospitalization for heart failure; RR：relative risk; HR: hazard rate
