# Supplementary material for: Sodium–Glucose Transporter 2 Inhibitors in Heart Failure: An Overview of Systematic Reviews
Source: J Cardiovasc Dev Dis. 2024 Jun 28;11(7):198. doi: 10.3390/jcdd11070198 (PMC11276734; doi:10.3390/jcdd11070198)
Supplement: Supplementary file 1 [file jcdd-11-00198-s001.zip › S2.pdf]

1.Usefulness of Sodium-Glucose Cotransporter 2 Inhibitors for Primary Prevention of Heart Failure in Patients With Type 2 Diabetes Mellitus
2.Sodium-glucose co-transporter 2 inhibitors and cardiovascular outcomes: A systematic review and meta-analysis
3.Sodium-glucose cotransporter 2 inhibitor effects on heart failure hospitalization and cardiac function: systematic review
4.Sodium glucose co-transporter 2 inhibitors in heart failure with preserved ejection fraction: a systematic review and meta-analysis
5.SGLT-2 INHIBITORS DEMONSTRATE CONSISTENT REDUCTION IN CARDIOVASCULAR MORTALITY AND HEART FAILURE HOSPITALIZATIONS AN UP-TO-DATE META-ANALYSIS
6.Robustness of outcomes in trials evaluating sodium-glucose co-transporter 2 inhibitors for heart failure
7.META-ANALYSIS EVALUATING THE EFFICACY OF SODIUM-GLUCOSE CO-TRANSPORTER-2 INHIBITORS IN SUBJECTS WITH HEART FAILURE WITH PRESERVED LEFT VENTRICULAR EJECTION FRACTION
8.Meta-Analysis Evaluating the Efficacy of Sodium-Glucose Co-Transporter-2 Inhibitors in Patients With Acute or Recently Decompensated Heart Failure
9.Meta-analysis of SGLT inhibitors on cardiovascular death or heart failure hospitalisation based on presence of type 2 diabetes, heart failure, or chronic kidney disease
10.Meta-Analysis Evaluating the Cardiovascular Efficacy of Sodium-Glucose Co-Transporter-2 Inhibitors in Patients With Heart Failure With Reduced Ejection Fraction According to Baseline Anemia
11.Meta-Analysis Evaluating Dapagliflozin in Heart Failure Reduced Ejection Fraction: A Focus on Left Ventricular Remodeling
12.A META-ANALYSIS ASSESSING THE EFFECT OF SODIUM-GLUCOSE CO-TRANSPORTER-2 INHIBITORS ON SURROGATE ENDPOINTS IN PATIENTS WITH HEART FAILURE WITH REDUCED EJECTION FRACTION
13.Kidney function after initiation and discontinuation of empagliflozin in heart failure patients with and without type 2 diabetes: insights from the emperial trials
14.Impact of Dapagliflozin on Mortality in Patients With Heart Failure and Reduced Ejection Fraction: A Meta-analysis
15.[Heart failure protection by SGLT2 inhibitors in patients with type 2 diabetes mellitus: evidence and possible mechanisms : A systematic review]
16.Heart failure hospitalization with SGLT-2 inhibitors: a systematic review and meta-analysis of randomized controlled and observational studies
17.Efficacy and safety of SGLT2 inhibitors in patients with heart failure A protocol for systematic review and meta-analysis
18.Efficacy and safety of dapagliflozin in the treatment of chronic heart failure A protocol for systematic review and meta-analysis
19.Effects of sodium-glucose cotransporter type 2 inhibitors on cardiovascular, renal, and safety outcomes in patients with cardiovascular disease: a meta-analysis of randomized controlled trials

20.Effects of SGLT-2 inhibitors on health-related quality of life and exercise capacity in heart failure patients with reduced ejection fraction: A systematic review and meta-analysis
21.EFFECTS OF CANAGLIFLOZIN ON CARDIOVASCULAR OUTCOMES AND ALL CAUSE MORTALITY: A REVIEW AND META-ANALYSIS
22.Effect of sodium-glucose cotransporter 2 inhibitors on cardiovascular and kidney outcomes -Systematic review and meta-analysis of randomized placebo-controlled trials
23.Effect of sodium-glucose cotransporter 2 inhibitors on cardiac structure and function in type 2 diabetes mellitus patients with or without chronic heart failure: a meta-analysis
24.Effect of sodium-glucose cotransporter-2 inhibitors on blood pressure in patients with heart failure: a systematic review and meta-analysis
25.Effect of sodium glucose cotransporter 2 inhibitors on cardiac function and cardiovascular outcome: a systematic review
26.Effect of empagliflozin in patients with heart failure across the spectrum of left ventricular ejection fraction
27.Effect of Dapagliflozin on Cause-Specific Mortality in Patients With Heart Failure Across the Spectrum of Ejection Fraction: a Participant-Level Pooled Analysis of DAPA-HF and DELIVER
28.Do SGLT-2 inhibitors exhibit similar cardiovascular benefit in patients with heart failure with reduced or preserved ejection fraction?
29.Dapagliflozin across the range of ejection fraction in patients with heart failure: a patient-level, pooled meta-analysis of DAPA-HF and DELIVER
30.Association of Sodium-Glucose Cotransporter 2 Inhibitors With Cardiovascular Outcomes in Patients With Type 2 Diabetes and Other Risk Factors for Cardiovascular Disease: A Meta-analysis
31.Association of SGLT2 inhibitors with arrhythmias and sudden cardiac death in patients with type 2 diabetes or heart failure: A meta-analysis of 34 randomized controlled trials
32.Association of Empagliflozin Treatment With Albuminuria Levels in Patients With Heart Failure: a Secondary Analysis of EMPEROR-Pooled
33.Association of Eligibility for a Sodium-Glucose Cotransporter 2 Inhibitor and Cardiovascular Events in Patients With Atrial Fibrillation
34.Sodium-glucose co-transporter 2 inhibitors and cardiovascular outcomes: A systematic review and meta-analysis
35.Effects of sodium glucose cotransporter type 2 inhibitors on heart failure
36.Generalizability of sodium-glucose co-transporter-2 inhibitors cardiovascular outcome trials to the type 2 diabetes population: a systematic review and meta-analysis
37.Meta-analysis addressing the impact of sodium-glucose Co-transporter-2 inhibitors on the risk for atrial fibrillation among individuals with heart failure with preserved ejection fraction
38.Meta-Analysis of Sodium-Glucose Cotransporter 2 Inhibitors in Heart Failure

Patients Without Diabetes
39.Sodium-Glucose Cotransporter-2 Inhibitors (SGLT-2i) Reduce Hospitalization for Heart Failure Only and Have No Effect on Atherosclerotic Cardiovascular Events: A Meta-Analysis
40.Sodium-glucose cotransporter 2 inhibitor effects on heart failure hospitalization and cardiac function: systematic review
41.Meta analysis of the effect of dapagliflozin on blood glucose and cardiovascular risk factors in patients with type 2 diabetes
42.Meta analysis of the effect of dapagliflozin on cardiovascular safety in patients with type 2 diabetes
43.Meta analysis and sequential trial analysis of the effect of SGLT2 inhibitor on cardiovascular adverse events in type 2 diabetes patients with cardiovascular disease
44.Treatment of patients with chronic heart failure with dapagliflozin：systematic review and meta analysis
45.Meta analysis of the efficacy and safety of dapagliflozin in the treatment of chronic heart failure
46.Updated Meta-Analysis Evaluating the Beneficial Effects of Sodium-Glucose Co-Transporter-2 Inhibitors in Patients With Heart Failure
47.Updated Meta-analysis Assessing the Effect of Sodium-Glucose Co-transporter-2 Inhibitors on Surrogate End points in Patients With Heart Failure With Reduced Ejection Fraction
48.A Meta-Analysis of the Sodium-Glucose Cotransporter 2 Inhibitors in Patients With Heart Failure and Preserved Ejection Fraction
49.Use of SGLT2 inhibitors and the incidence of urinary tract infections in patients with heart failure: a pooled analysis of four trials including 26.838 patients
50.Effects of canagliflozin on cardiovascular death and hospitalisation for heart failure by eGFR: integrated analyses of the CANVAS Program and CREDENCE
51.Cardiovascular Outcomes of SGLT2 Inhibitors Across Sex or Race in Patients with/out Diabetes and Heart Failure
52.Efficacy and safety of sodium-glucose cotransporter 2 inhibitors initiation in patients with acute heart failure, with and without type 2 diabetes: a systematic review and meta-analysis
53.Benefit of sodium-glucose cotransporter-2 inhibitors on survival outcome is related to the type of heart failure: A meta-analysis
54 The effect of sodium-glucose cotransporter-2 inhibitors on cardiac structure remodeling and function: A meta-analysis of randomized controlled trials
55.Dapagliflozin across the range of ejection fraction in patients with heart failure: a patient-level, pooled meta-analysis of DAPA-HF and DELIVER
56.Effect of sodium-glucose cotransporter-2 inhibitors on blood pressure in patients with heart failure: a systematic review and meta-analysis
57.The Effect of Sodium-Glucose Co-Transporter 2 Inhibitors on Stroke and Atrial Fibrillation: A Systematic Review and Meta-Analysis
58.Efficacy of sodium glucose cotransporter 2 inhibitors for heart failure with preserved and mildly reduced ejection fraction: a systematic review and meta-analysis

59.Optimal Pharmacologic Treatment of Heart Failure with Preserved and Mildly Reduced Ejection Fraction: A Meta-analysis
60.Robustness of outcomes in trials evaluating sodium-glucose co-transporter 2 inhibitors for heart failure
61.SGLT2 Inhibitors Reduce HeartFailure Hospitalization and Cardiovascular Death: Clarity and Consistency.
62.SGLT-2 INHIBITORS ARE EFFICACIOUS IN HIGH-RISK SUBGROUPS OF PATIENTS WITH HEART FAILURE WITH MILDLY REDUCED OR PRESERVED EJECTION FRACTION
63.Sodium Glucose Cotransporter 2 Inhibitors, Amputation Risk, and Fracture Risk
Systematic review of sodium-glucose cotransporter 2 inhibitors: a hopeful prospect in tackling heart failure-related events
64."Sodium-Glucose Cotr Sodium-Glucose Cotransporter 2 Inhibit ter 2 Inhibitors Among Hear ors Among Heart FailureWith Mildly Reduced and Pr With Mildly Reduced and Preserved Ejection F ed Ejection Fraction"
65.Effects of sodium-glucose cotransporter type 2 inhibitors on cardiovascular, renal, and safety outcomes in patients with cardiovascular disease: a meta-analysis of randomized controlled trials
66.Do SGLT-2 inhibitors exhibit similar cardiovascular benefit in patients having reduced ejection fraction heart failure with type 2 diabetes, prediabetes and normoglycemia?
