# Supplementary material for: Sodium–Glucose Transporter 2 Inhibitors in Heart Failure: An Overview of Systematic Reviews
Source: J Cardiovasc Dev Dis. 2024 Jun 28;11(7):198. doi: 10.3390/jcdd11070198 (PMC11276734; doi:10.3390/jcdd11070198)
Supplement: Supplementary file 1 [file jcdd-11-00198-s001.zip › S4.pdf]

Table S2 The results of AMSTAR2

Review	Q1	Q2	Q3	Q4	Q5	Q6	Q7	Q8	Q9	Q10	Q11	Q12	Q13	Q14	Q15	Q16	Outcome
Wang et al,2021[29]	Y	N	N	PY	Y	N	N	N	Y	N	Y	N	N	Y	Y	N	CL
Athiyaman et al,2022[30]	PY	N	N	PY	N	N	N	N	Y	N	N	N	N	Y	N	Y	CL
Camilli et al,2022[31]	Y	N	N	N	Y	Y	N	Y	Y	N	Y	Y	Y	Y	N	N	CL
Ahmad etal,2021[32]	PY	PY	N	Y	Y	Y	N	Y	Y	Y	Y	Y	Y	Y	N	Y	CL
Kumar et al,2020[33]	Y	N	N	PY	Y	Y	N	Y	N	N	Y	N	N	N	N	N	CL
Pandey et al,2022[34]	Y	PY	N	Y	Y	Y	N	Y	Y	N	Y	Y	Y	Y	N	Y	CL
Fukuta et al,2022[35]	Y	Y	N	Y	N	Y	N	Y	Y	Y	Y	N	Y	Y	N	Y	CL
Zou et al,2022[37]	Y	Y	N	Y	Y	Y	N	Y	Y	Y	Y	Y	Y	Y	Y	Y	L
Shrestha et al,2021[38]	Y	PY	N	PY	N	N	N	PY	Y	N	Y	N	N	Y	Y	N	CL
Cao et al,2022[39]	Y	PY	N	Y	Y	Y	N	Y	Y	Y	Y	Y	Y	Y	Y	Y	L
Vukadinovi´c et al,2022[41]	Y	PY	N	Y	Y	Y	N	Y	Y	Y	Y	Y	Y	Y	Y	Y	L
Vaduganathan et al,2022[42]	Y	Y	N	Y	Y	Y	N	Y	Y	Y	Y	Y	Y	Y	Y	Y	L
Wu et al,2022[43]	Y	N	N	Y	Y	Y	N	Y	Y	N	Y	Y	Y	Y	Y	N	CL
Zannad et al,2020[45]	Y	PY	N	N	N	Y	N	Y	N	Y	Y	N	N	Y	N	Y	CL
Cardoso et al,2021[46]	Y	PY	N	Y	N	Y	N	Y	Y	Y	Y	Y	Y	Y	Y	Y	L
Younes et al,2022[47]	PY	Y	N	Y	Y	N	N	PY	N	Y	Y	N	N	Y	N	Y	CL
Tsampasian et al,2021[48]	Y	Y	N	PY	Y	Y	N	PY	Y	Y	Y	Y	Y	Y	Y	Y	L
Bazoukis et al,2021[50]	Y	PY	N	Y	Y	Y	N	PY	Y	N	N	Y	Y	N	Y	N	CL
Butler et al,2020[52]	Y	Y	N	Y	Y	Y	N	Y	Y	Y	Y	Y	Y	Y	Y	N	L
Li et al,2021[53]	Y	PY	N	PY	Y	Y	N	Y	Y	Y	Y	Y	Y	Y	Y	Y	L
Zheng et al,2020[54]	PY	PY	N	Y	Y	Y	N	PY	Y	Y	Y	Y	Y	Y	N	Y	CL
Zhai et al,2021[55]	Y	PY	N	Y	Y	Y	N	Y	Y	N	Y	Y	Y	Y	Y	N	L
Chambergo-Michilot et al,2020[56]	Y	Y	N	PY	Y	Y	N	Y	Y	Y	Y	Y	Y	Y	N	Y	CL
Zhou et al,2022[57]	Y	Y	N	Y	N	Y	Y	Y	Y	Y	Y	Y	Y	Y	Y	Y	H
Lu et al,2021[58]	Y	PY	N	Y	Y	Y	N	Y	Y	Y	Y	Y	Y	Y	Y	Y	L
Cai et al,2021[59]	Y	Y	N	Y	Y	Y	N	Y	Y	Y	Y	Y	Y	Y	Y	Y	L
Singh et al,2021[61]	Y	PY	N	PY	Y	Y	N	Y	Y	Y	Y	N	N	Y	Y	Y	CL
Sfairopoulos et al.2022[64]	Y	PY	N	Y	Y	Y	N	Y	Y	Y	Y	Y	Y	Y	Y	Y	L
Gager et al.2021[62]	Y	PY	N	PY	PY	N	Y	N	N	Y	Y	N	N	Y	Y	Y	L
Treewaree et al.2023[49]	Y	Y	N	Y	Y	Y	N	Y	Y	N	Y	Y	Y	Y	Y	N	L
Zhao et al,2022[63]	Y	PY	N	Y	Y	Y	N	PY	Y	Y	Y	Y	Y	Y	N	Y	CL
Chen et al,2022[60]	Y	Y	N	Y	N	Y	N	Y	Y	N	Y	Y	Y	Y	Y	Y	L
De Marzo et al,2023[51]	N	Y	N	Y	Y	Y	N	Y	Y	N	Y	Y	Y	N	Y	Y	L
Oates et al ,2023[44]	Y	N	N	Y	N	Y	N	Y	N	N	Y	N	N	Y	Y	N	CL
Ismayl etal,2023[40]	Y	PY	N	Y	N	Y	N	Y	Y	N	Y	Y	Y	Y	Y	N	L
Ouyang et al,2023[36]	Y	N	N	Y	N	Y	N	Y	Y	N	Y	N	Y	Y	Y	N	L

Q1: Did the research questions and inclusion criteria for the review include the components of PICO?

Q2: Did the report of the review contain an explicit statement that the review methods were established prior to the conduct of the review and did the report justify any significant

deviations from the protocol?

Q3: Did the review authors explain their selection of the study designs for inclusion in the review?

Q4: Did the review authors use a comprehensive literature search strategy?

Q5: Did the review authors perform study selection in duplicate?

Q6: Did the review authors perform data extraction in duplicate?

Q7: Did the review authors provide a list of excluded studies and justify the exclusions?

Q8: Did the review authors describe the included studies in adequate detail?

Q9: Did the review authors use a satisfactory technique for assessing the risk of bias (RoB) in individual studies that were included in the review?

Q10: Did the review authors report on the sources of funding for the studies included in the review?

Q11: If meta-analysis was performed, did the review authors use appropriate methods for statistical combination of results?

Q12: If meta-analysis was performed, did the review authors assess the potential impact of RoB in individual studies on the results of the meta-analysis or other evidence synthesis?

Q13: Did the review authors account for RoB in primary studies when interpreting/discussing the results of the review?

Q14: Did the review authors provide a satisfactory explanation for, and discussion of, any heterogeneity observed in the results of the review?

Q15: If they performed quantitative synthesis did the review authors carry out an adequate investigation of publication bias (small study bias) and discuss its likely impact on the

results of the review?

Q16：Did the review authors report any potential sources of conflict of interest, including any funding they received for conducting the review?

Y：every item is compliant PY：partially compliant with standard N：completely out of standard CL: critically low L:low H：high
