# Supplementary material for: Sodium–Glucose Transporter 2 Inhibitors in Heart Failure: An Overview of Systematic Reviews
Source: J Cardiovasc Dev Dis. 2024 Jun 28;11(7):198. doi: 10.3390/jcdd11070198 (PMC11276734; doi:10.3390/jcdd11070198)
Supplement: Supplementary file 1 [file jcdd-11-00198-s001.zip › S6.pdf]

Table S4 The result of ROBIS

Review	Phase 1	Phase 2				Phase 3
		Domain 1	Domain 2.	Domain 3.	Domain 4.	
Wang et al,2021[29]	☺	☺	☺	?	?	☺
Athiyaman et al,2022[30]	☺	☹	☹	?	☹	?
Camilli et al,2022[31]	☺	☹	☹	?	☹	☹
Ahmad etal,2021[32]	☺	☺	☺	?	☺	?
Kumar et al,2020[33]	☺	☺	☹	☹	?	?
Pandey et al,2022[34]	☺	☺	☹	☺	☹	☺
Fukuta et al,2022[35]	☺	☺	☹	?	☹	☺
Zou et al,2022[37]	☺	☺	☺	☺	☺	☺
Shrestha et al,2021[38]	☺	☺	☹	?	☹	☹
Cao et al,2022[39]	☺	☺	☹	?	☺	☺
Vukadinovi´c et al,2022[41]	☺	☹	☹	☺	☺	☺
Vaduganathan et al,2022[42]	☺	☺	☹	?	☺	☺
Wu et al,2022[43]	☺	☺	☹	☺	?	☺
Zannad et al,2020[45]	☺	☺	☹	☹	☺	?
Cardoso et al,2021[46]	☺	☺	☹	?	?	☺
Younes et al,2022[47]	☺	☺	☹	☹	?	?
Tsampasian et al,2021[48]	☺	☺	☹	?	☺	☺
Bazoukis et al,2021[50]	☺	☹	☹	?	?	☺
Butler et al,2020[52]	☺	☺	☺	?	☺	☺
Li et al,2021[53]	☺	☺	☹	?	☺	☺
Zheng et al,2020[54]	☺	☹	☹	☺	☺	☺
Zhai et al,2021[55]	☺	☺	☹	☺	☺	☺
Chambergo-Michilot et al,2020[56]	☺	☹	☹	☺	☺	☺
Zhou et al,2022[57]	☺	☺	☹	☺	☺	☺
Lu et al,2021[58]	☺	☺	☺	?	☺	☺
Cai et al,2021[59]	☺	☺	☺	?	☺	☺
Singh et al,2021[61]	☺	☺	☹	☺	☹	☹
Sfairopoulos et al.2022[64]	☺	☺	☺	?	?	☺
Gager et al.2021[62]	☺	☺	☹	☹	?	☹
Treewaree et al.2023[49]	☺	☺	☹	☺	?	☺
Zhao et al,2022[63]	☺	☹	☺	☺	?	☺
Chen et al,2022[60]	☺	☺	☺	☺	☺	☺
De Marzo et al,2023[51]	☹	☺	☹	?	☺	☺
Oates etal,2023[44]	☺	☺	☹	☹	☹	☹
Ismayl etal,2023[40]	☺	☺	?	?	☺	☺
Ouyang etal,2023[36]	☺	☺	☺	?	?	☺

☺: low risk;☹:high risk; ?: unclear risk
