# Supplementary material for: Sodium–Glucose Transporter 2 Inhibitors in Heart Failure: An Overview of Systematic Reviews
Source: J Cardiovasc Dev Dis. 2024 Jun 28;11(7):198. doi: 10.3390/jcdd11070198 (PMC11276734; doi:10.3390/jcdd11070198)
Supplement: Supplementary file 1 [file jcdd-11-00198-s001.zip › S9.pdf]

Table S7 Result Of Evidence Quality （GRADE）

Outcome	Systematic review	Certainty assessment							Certainty
		Relative effect （95%CI）	studies（patiens）	Risk of bias	Inconsistency	Indirectness	Imprecision	Other considerations	
Atrial fibrillation	Sfairopoulos et al,2022[64]	RR 0.62 （0.44,0.86）	6（142）	not serious	not serious	not serious	not serious	not serious	High
Atrial fibrillation	Ouyang et al,2023[36]	RR 0.92(0.8,1,06)	10(727)	not serious	not serious	not serious	serious(g)	not serious	Moderate
Atrial flutter	Sfairopoulos et al,2022[64]	RR 0.85 （0.09,7.92）	2（25）	not serious	serious(f)	not serious	serious(g,h)	not serious	low
Ventricular arrhythmia	Oates et al, 2023[44]	RR 1.03(0.83,1.29)	8(301)	not serious	not serious	not serious	serious(g)	not serious	Moderate
Atrial arrhythmias	Oates et al,2023[44]	RR 0.91(0.77,1.09)	8(496)	not serious	not serious	not serious	not serious	not serious	High

f: I^2>50% or the heterogeneity test P was very small; g: confidence interval contains invalid line; h: sample size is too small; RR: relative risk
